# Chronic recurrent multifocal osteomyelitis in a patient with Familial Mediterranean Fever

**DOI:** 10.1186/1546-0096-13-S1-P104

**Published:** 2015-09-28

**Authors:** A Daia, V Kini, RZ Taha, H El-Shanti, B Fathalla

**Affiliations:** 1 Hamad General Hospital, Pediatrics, Doha, Qatar; 2 Hamad General Hospital, Radiology, Doha, Qatar; 3 Qatar Biomedical Research Institute, Medical Genetics Center, Doha, Qatar; 4 Hamad General Hospital, Pediatrics / Rheumatology, Doha, Qatar

## Introduction

Patients with more than one autoinflammatory disorder are rarely reported in the literature [1]. Additionally, rare reports suggest that *MEFV* mutations might be associated with atypical manifestations for familial Mediterranean fever (FMF), such as isolated recurrent muscle pain in one patient [2] and chronic recurrent multifocal osteomyelitis (CRMO) responsive to colchicine in another patient heterozygote for E148Q-P369S-R408Q *MEFV* complex allele [3]. We report a unique case of a patient with classic FMF developing CRMO later in the disease and discuss genetic test results, imaging features, and response to treatment.

## Methods

A retrospective review of medical records was conducted and one radiologist reviewed all imaging studies. The molecular genetic testing for *MEFV*, *PSTPIP1*, *IL1RN*, *PSTPIP2*, and *LPIN2* was done by direct resequencing of the entire coding sequence and the splice sites.

## Case report

A 13 years old Arabic female diagnosed with FMF at the age of 10 years based on recurrent 3-days episodes of abdominal pain every 2-3 weeks occasionally associated with fever but without chest pain, rash, or arthralgia for at least one year. Genetic testing revealed heterozygote p.M694V *MEFV* mutation. She was responsive to daily colchicine at 0.5 mg. At the age of 12 years she developed severe acute dorsal and lower back pain, and arthralgia of the lower extremities. MRI imaging demonstrated both epiphyseal and metaphyseal involvement of left femoral head, right proximal tibial metaphysis, right distal tibial metaphysis and epiphysis, proximal epiphysis of first metatarsal bone, bilateral knee synovitis with effusion, and D-11 vertebra and left sacroiliac disease. She received one cycle of monthly pamidronate for three months in addition to naproxen with clinical and radiological improvement as demonstrated in repeated MRI at 6 months intervals. Imaging showed full resolution of bone lesions without recurrences or new lesions but continue to demonstrate active knees arthritis at 24 months follow-up. Spine MRI showed resolution of inflammatory changes noted at D-11 with shortening of vertebral body height and end plate erosions. She developed pustulosis palmoplantaris at the age of 13 years. She is currently on colchicine 1 mg twice daily due to elevated serum amyloid A level despite being asymptomatic. Methotrexate and infliximab will be started due to persistent arthritis. HLA-B27 test was negative and testing for *LPIN2*, *IL1RN*, *PSTPIP 1* and *PSTPIP 2* did not detect any mutation in the coding region or splice sites.

## Conclusion

The unique clinical course of our patient demonstrates the importance of considering a coexisting autoinflammatory disorder when patients present with atypical features of an already established and diagnosed autoinflammatory disease.

## Consent to publish

Written informated consent for publication of their clinical details was obtained from the patient/parent/guardian/relative of the patient.
